# Experimental multistable states for small network of coupled pendula

**DOI:** 10.1038/srep29833

**Published:** 2016-07-21

**Authors:** Dawid Dudkowski, Juliusz Grabski, Jerzy Wojewoda, Przemyslaw Perlikowski, Yuri Maistrenko, Tomasz Kapitaniak

**Affiliations:** 1 Division of Dynamics, Technical University of Lodz, Stefanowskiego 1/15, 90-924 Lodz, Poland; 2 Institute of Mathematics and Centre for Medical and Biotechnical Research, National Academy of Sciences of Ukraine, Tereshchenkivska st. 3, 01030 Kyiv, Ukraine; 3 Institut fur Theoretische Physik, Technische Universitat Berlin, Hardenbergstrasse 36, 10623 Berlin, Germany

## Abstract

Chimera states are dynamical patterns emerging in populations of coupled identical oscillators where different groups of oscillators exhibit coexisting synchronous and incoherent behaviors despite homogeneous coupling. Although these states are typically observed in the large ensembles of oscillators, recently it has been shown that so-called weak chimera states may occur in the systems with small numbers of oscillators. Here, we show that similar multistable states demonstrating partial frequency synchronization, can be observed in simple experiments with identical mechanical oscillators, namely pendula. The mathematical model of our experiment shows that the observed multistable states are controlled by elementary dynamical equations, derived from Newton’s laws that are ubiquitous in many physical and engineering systems. Our finding suggests that multistable chimera-like states are observable in small networks relevant to various real-world systems.

Chimera states correspond to the spatiotemporal patterns in which synchronized and phase locked oscillators coexist with desynchronized and incoherent ones^1^,^2^,^3^,^4^,^5^,^6^,^7^,^8^,^9^,^10^,^11^,^12^,^13^,^14^,^15^,^16^,^17^,^18^,^19^,^20^,^21^,^22^,^23^,^24^,^25^. These patterns have been reported both in simulations^1^,^2^,^3^,^4^,^5^,^6^,^7^,^8^,^9^,^10^,^11^,^12^,^13^,^14^,^15^,^16^,^17^,^18^,^26^ and experiments^19^,^20^,^21^,^22^,^23^,^24^,^25^ of the large networks of coupled oscillators with a variety of topologies. Recently, Ashwin & Burylko^27^ defined a *weak* chimera state as one referring to a trajectory in which two or more oscillators are frequency synchronized and one or more oscillators drift in phase and frequency with respect to the synchronized group. It has been found that these states can be observed in small networks as few as 4 phase oscillators (two groups of in-phase and antiphase oscillators)^27^,^28^,^29^.

Up to now weak chimera states in small networks have been reported in simulation and theory of coupled phase oscillators. Here, we show that similar multistable chimera-like states can be observed experimentally in small networks of more general oscillators. As a proof of concept, we use the network of four coupled externally excited double pendula. Each pendulum is characterized by the coexistence of rotational or oscillatory periodic solutions of different frequencies. We argue that such multistability implies the occurrence of these states and present evidence that they can persist for a positive measure set of coupling strength.

We consider the system of *4* identical coupled double pendula arranged into a cross configuration, as shown in Fig. 1(a) The lower pendula’s bobs (marked with symbols *II*_*i*_, *i* = 1, 2, 3, 4) can rotate or oscillate around their horizontal axes at points *D*_1_, *D*_2_, *D*_3_, *D*_4_. The displacements of these bobs are given by *φ*_*i*2_(*t*). Lower bobs are connected to the upper bobs by the rotational pivots at *D*_*i*_. The upper bobs (*I*_*i*_) can only oscillate around the horizontal axes marked by *A*_1_, *A*_2_, *A*_3_ and *A*_4_ and located on the base *III*. One of the bob’s ends is connected to the base by the rotational pivot at *A*_*i*_ and the second ends are suspended on the springs characterized by the stiffness coefficient *k*_*s*_. The displacements of upper bobs are given by *φ*_*i*1_(*t*). The upper bobs *I*_*i*_ of length *η*_*1*_ have mass *m*_*1*_ and moment of inertia *J*_*1*_ while the lower bobs *II*_*i*_ of length *η*_*2*_ have mass *m*_*2*_ and moment of inertia *J*_*2*_. The detailed geometry is shown in Fig. 1(b). The viscoelastic damping is assumed in the pivots at *D*_*i*_ (with damping coefficient *c*_*c*_) and *A*_*i*_ (with damping coefficient *k*_*c*_). The base, mounted on the shaker, is excited in the vertical direction by the kinematic displacement, *y* = *A*cos *ωt*. The upper pendula’s bobs are coupled to the nearest neighbor by the plane springs (with stiffness coefficient α) shown in green. The similar system in which pendula have not been coupled i.e., the system without plane springs has been considered by Strzalko *et al*.^30^.

The dynamics of the system of Fig. 1(a,b) can be analyzed using the equations of motion (see Methods).

## Results

In the absence of coupling (when one removes green planar springs and thus, coupling parameter *α* = 0 in Equation(1) in Methods) it is possible to identify excitation parameters (*A* and ω) for which each double pendulum exhibits multistability. In Fig. 2 we present regions of existence of various *N:M* , where *N* is the number of rotation/oscillation of lower pendulum *II*_1–4_ and *M* is the number of periods of excitations, eg., 1:1 means that pendula *II*_1–4_ oscillate or rotate with the frequency of the excitation *ω*, 2:1 (pendula *II*_1–4_ oscillate or rotate with the frequency of the excitation ½ *ω*), etc. One can identify six main regions, indicated from 1 to 6 in Fig. 2, in which the excited double pendulum is multistable. In region 1 three solutions exist: 1:1 rotations (above the green line), 1:4 oscillations (between the dashed red lines) and 1:2 rotations (between solid black lines). Region 2 is characterized by the co-existence of four solutions: 1:1 rotations (above the green line), 1:4 oscillations (between the dashed red lines), 1:2 rotations (between solid black lines) and 3:6 rotations (between solid orange lines). Four solutions are stable also in region 3:1:1 rotations (above the green line), 1:6 oscillations (between the dashed black lines), 1:2 rotations (between solid black lines), 1:3 rotations (between solid yellow lines). Three solutions: 1:1 rotations (above the green line), 1:6 oscillation (between dashed black lines), 1:2 rotation (between solid black lines) can be observed in region 4. Region 5 is another example of the co-existence of three solutions: 1:1 rotations (above the green line), 1:2 rotations (between solid black lines), 1:3 rotation (between solid yellow lines). Finally in region 6 we observe four solutions: 1:1 rotations (above green line), 1:4 oscillations (between the dashed black lines), 3:6 rotations (between solid orange lines).

In regions 1–6 each of four uncoupled double pendula can exhibit *M* (equal to 3 or 4) various independent dynamical responses, i.e., 1:1, 2:1 or 3:1 rotational and oscillatory solutions. The set of 4 pendula is characterized by 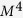 configurations. One can see that the number of configurations grows exponentially with the number of pendula (i.e., in the case of *n* pendula we have 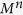 configurations) so there is spatial chaos^31^ in an uncoupled system. For sufficiently small coupling one can observe multistable chimera-like states which persist over the wide range of system parameters and can be captured experimentally. These states coexist with various cases of complete, phase and cluster synchronous states.

Experimentally observed multistable chimera-like states are illustrated in Fig. 3(a–f). Upper images present general view of the pendula’s configurations while lower plots show time series of the lower pendula bobs. The figures present a kind of a stroboscope type images of the pendula motion in different cases. All experiments have been recorded using Vision Research Phantom v711 high speed camera. Typical recording speed was 1000 frames per second (fps) and for the purpose of a still photograph visualization a set of 5 of them every fifth frame: 5 × 0.001 = 0.005 seconds have been chosen. Then, the images were combined to a single image presenting all chosen images overlaid with the assumed transparency level. The wider area covered by the set of frozen images of each pendulum, the faster speed of its rotation or oscillation and vice verse. In Fig. 3(a–d) we show multistable states in which all the pendula rotate (*A* = 0.01[m], ω = 18π [rad/s]–region 5 of Fig. 2). In Fig. 3(a) pendula 1 and 2 rotate with frequency 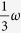 and pendula 3 and 4 with frequency 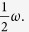 Pendula 3 and 4 are in antiphase to each other (see movie W1). The case in which pendula 1, 3 and 4 rotate with frequency 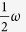 and pendulum 2 with frequency 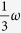 is shown in Fig. 3(b). Pendula 1 and 4 are synchronized in phase and pendulum 3 is in antiphase to pendula 1 and 4 (see movie W2). Configuration of Fig. 3(c) presents the case when pendulum 1 rotates with a frequency 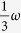, pendula 2 and 3 with frequency 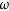 and pendulum 4 with frequency 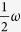. Pendula 2 and 3 are synchronized (see movie W3). Figure 3(d) shows the configuration in which pendula 1, 2 and 4 rotate with frequency 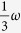 and pendulum 3 with frequency 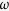. Pendula 1 and 2 are synchronized in phase (see movie W4).

In Fig. 3(e,f) we observe multistable states in which the pendula show both rotational and oscillatory behavior (*A* = 0.005[m], ω = 10π [rad/s]–region 1 of Fig. 2). Figure 3(e) shows the chimera-like state in which pendula 1, 3 and 4 rotate with frequency 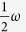 while pendulum 2 oscillates with frequency 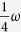. Pendula 1 and 4 are synchronized in phase (see movie W5). The chimera-like state shown in Fig. 3(f) is characterized by 3 rotating and one oscillating pendula. Pendula 1 and 4 rotate with the frequency ω and are synchronized in phase. Pendula 2 and 3 respectively rotate with frequency 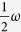 and oscillate with frequency 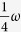 (see movie W6).

The presented multistable states coexist with various synchronous states. Movies W7, W8, W9 present the case of the complete synchronization of all pendula in rotational motion (W7), the case when all pendula oscillate with frequency ω and pendula 2, 3, 4 are synchronized in phase and pendulum 1 is in antiphase to them (W8) and the case when all pendula oscillate with the frequency ω and pendula 1, 3 and 2, 4 create two clusters of phase synchronized pendula respectively. These clusters are in antiphase to each other (W9).

In conclusion, we have constructed the simple experimental setup to explore the spatio-temporal dynamics of the small network of the locally coupled pendula. The nodes in the network are externally excited double pendula. Despite a small number of nodes, namely 4, we observe the formation of spatio-temporal patterns of multistable chimera-like states. This behavior is observed experimentally, confirmed in numerical simulations, persistent over a positive measure set of system parameters and seems to be characteristic for the small networks of coupled multistable general oscillators relevant to various real-world systems.

## Methods

**Figure 1:**
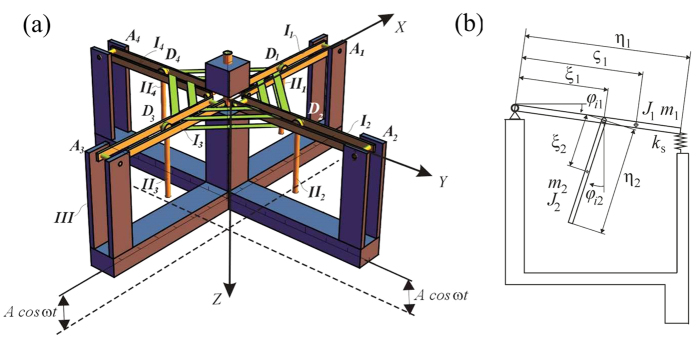
(**a**) Model of a set of (***N*** = 4) double pendula located at an oscillating platform, (**b**) geometry of *i*-th double pendulum.

The dynamics of the system of coupled pendula shown in Fig. 1(a) is given by:


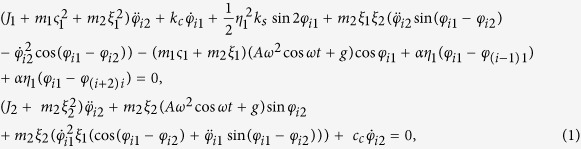


where *i* = 1, 2, 3, 4.

### Numerical simulations

We used the following parameter values: *J*_1_ = 4.521 × 10^−3^[kgm^2^], *J*_2_ = 2.908 × 10^−5^[kgm^2^], *m*_1_ = 0.5562[kg], *m*_2_ = 0.0166[kg], *ξ*_1_ = 0.153[m], *ξ*_2_ = 0.096[m], 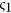 = 0.180[m], *η*_1_ = 0.315[m], *η*_2_ = 0.145[m], *k*_*s*_ = 6850[N/m] 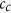 = 0.5 × 10^−4^[Nms] and *k*_*c*_ = 0.050[Nms]. The parameters values used in experiment have been independently measured.

**Figure 2:**
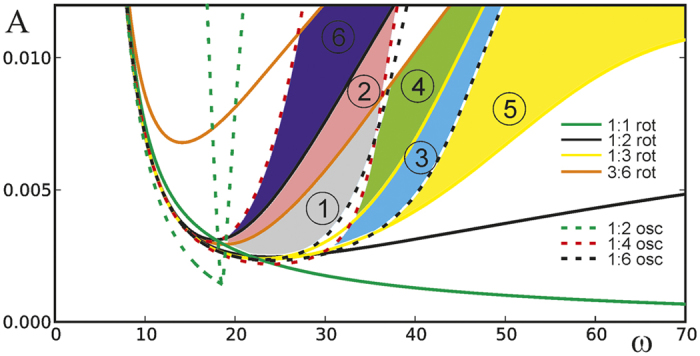
Regions of existence of different types of rotational or oscillatory responses of the uncoupled pendulum in the space of parameters *A* and *ω*. In regions 1–6 double pendulum is multi stable with co-existing solutions of different frequencies. In these regions for α > 0 the multistable chimera-like states can be observed.

**Figure 3:**
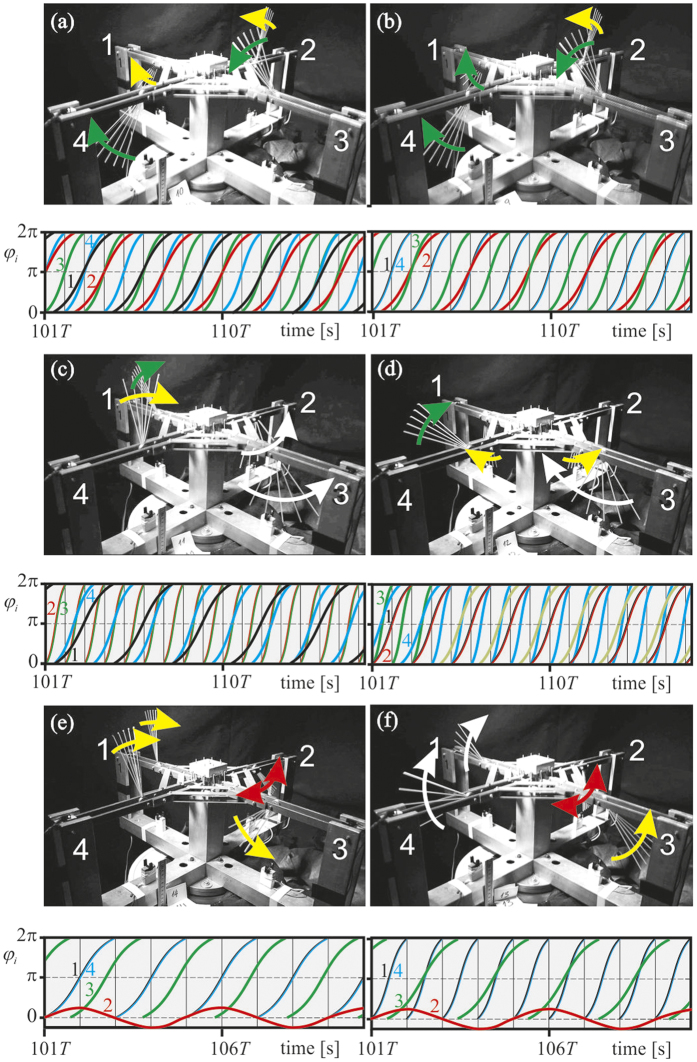
Experimentally observed multistable chimera-like states: (**a–d**) *A* = 0.01[m], ω = 18π [rad/s] (region 5 of Fig. 2), (**e,f**) *A* = 0.005[m], ω = 10π [rad/s] (region 1 of Fig. 2); (**a**) pendula 1 and 2 rotate with frequency 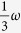, pendula 3 and 4 with frequency 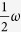, (**b**) pendula 1, 3 and 4 rotate with frequency 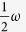 and pendulum 2 with frequency 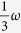, (**c**) pendulum 1 rotates with frequency 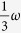, pendula 2 and 3 with frequency 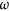 and pendulum 4 with frequency 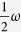, (**d**) pendula 1, 2 and 4 rotate with frequency 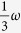 and pendulum 3 with frequency 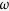, (**e**) pendula 1, 3 and 4 rotate with frequency 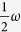, pendulum 2 oscillates with frequency 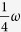, (**f**) pendula 1 and 4 rotate with frequency ω, pendulum 2 rotates with frequency 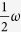 and pendulum 3 oscillates with the frequency 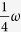.

Eqs (1) have been integrated by the 4^th^ order Runge-Kutta method. Bifurcation curves in Fig. 2 have been calculated using path following method AUTO^32^.

### Experimental observations

In our experiments, the rig with four coupled double pendula has been mounted on the shaker LDS V780 Low Force Shaker (basic data are as follows: sine force peak 5120[N], max random force (rms) 4230[N], max acceleration sine peak *g*_*n*_ = 111 g [m/s^2^], system velocity sine peak 1.9[m/s], displacement pk-pk *g*_*n*_ = 25.4[mm], moving element mass 4.7[kg]). The shaker introduces practically kinematic periodic excitation 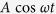, where *A* and *ω* are the amplitude and the frequency of the excitation, respectively. All experiments were recorded at motion videos taken by Vision Research Phantom v711 high speed camera. Typical recording speed used was 1000 frames per second (fps). Different random initial conditions have been given to each pendulum.

## Additional Information

**How to cite this article**: Dudkowski, D. *et al*. Experimental multistable states for small network of coupled pendula. *Sci. Rep.* **6**, 29833; doi: 10.1038/srep29833 (2016).

## Supplementary Material

Supplementary Video 1

Supplementary Video 2

Supplementary Video 3

Supplementary Video 4

Supplementary Video 5

Supplementary Video 6

Supplementary Video 7

Supplementary Video 8

Supplementary Video 9

Supplementary Information

## References

[b1] Kuramoto Y. & Battogtokh D. Coexistence of coherence and incoherence in nonlocally coupled phase oscillators. Nonlinear Phen. Complex Syst. 5, 380–385 (2002).

[b2] Abrams D. M. & Strogatz S. H. Chimera states for coupled oscillators. Phys. Rev. Lett. 93, 174102 (2004). 15525081 10.1103/PhysRevLett.93.174102

[b3] Abrams D. M., Mirollo R., Strogatz S. H. & Wiley D. A. Solvable model for chimera states of coupled oscillators. Phys. Rev. Lett. 101, 084103 (2008). 18764617 10.1103/PhysRevLett.101.084103

[b4] Martens E. A., Laing C. R. & Strogatz S. H. Solvable model of spiral wave chimeras. Phys. Rev. Lett. 104, 044101 (2010). 20366714 10.1103/PhysRevLett.104.044101

[b5] Motter A. E. Nonlinear dynamics: Spontaneous synchrony breaking. Nat. Phys. 6, 164–165 (2010).

[b6] Omelchenko I., Maistrenko Y. L., Hövel P. & Schöll E. Loss of coherence in dynamical networks: Spatial chaos and chimera states. Phys. Rev. Lett. 106, 234102 (2011). 21770506 10.1103/PhysRevLett.106.234102

[b7] Omelchenko I., Riemenschneider B., Hövel P., Maistrenko Y. L. & Schöll E. Transition from spatial coherence to incoherence in coupled chaotic systems. Phys. Rev. E 85, 026212 (2012). 10.1103/PhysRevE.85.026212 22463304

[b8] Laing C. R. The dynamics of chimera states in heterogeneous Kuramoto networks. Physica D 238, 15691588 (2009).

[b9] Laing C. R. Chimeras in networks of planar oscillators. Phys. Rev. E 81, 066221 (2010). 10.1103/PhysRevE.81.066221 20866515

[b10] Laing C. R. Fronts and bumps in spatially extended Kuramoto networks. Physica D 240, 1960–1971 (2011).

[b11] Martens E. A. Bistable chimera attractors on a triangular network of oscillator populations. Phys. Rev. E 82, 016216 (2010). 10.1103/PhysRevE.82.016216 20866716

[b12] Martens E. A. Chimeras in a network of three oscillator populations with varying etwork topology. Chaos 20, 043122 (2010). 21198092 10.1063/1.3499502

[b13] Wolfrum M. & Omel’chenko O. E. Chimera states are chaotic transients. Phys. Rev. E 84, 015201 (2011). 10.1103/PhysRevE.84.015201 21867244

[b14] Sethia G. C., Sen A. & Atay F. M. Clustered chimera states in delay-coupled oscillator systems. Phys. Rev. Lett. 100, 144102 (2008). 18518036 10.1103/PhysRevLett.100.144102

[b15] Waller I. & Kapral R. Spatial and temporal structure in systems of coupled nonlinear oscillators. Phys. Rev. A 30, 20472055 (1984).

[b16] Zakharova A., Kapeller M. & Scholl E. Chimera death: Symmetry breaking in dynamical networks. Phys. Rev. Lett. 112, 154101 (2014). 24785041 10.1103/PhysRevLett.112.154101

[b17] Jaros P., Maistrenko Yu. & Kapitaniak T. Chimera states on the route from coherence to rotating waves. Phys. Rev. E 91, 022907 (2015). 10.1103/PhysRevE.91.022907 25768569

[b18] Dudkowski D., Maistrenko Yu. & Kapitaniak T. Different types of chimera states: An interplay between spatial and dynamical chaos. Phys. Rev. E. 90, 032920 (2014). 10.1103/PhysRevE.90.032920 25314517

[b19] Hagerstrom A. M. . Experimental observations of chimera states in coupled-map lattices. Nat. Phys. 8, 658 (2012).

[b20] Tinsley M. R., Nkomo S. & Showalter K. Chimera and phase-cluster states in populations of coupled chemical oscillators. Nat. Phys. 8, 662 (2012). 10.1103/PhysRevLett.110.244102 25165927

[b21] Martens E. A., Thutupalli S., Fourriere A. & Hallatschek O. Chimera states in mechanical oscillator networks. Proc. Nat. Acad. Sciences 110, 10563 (2013). 10.1073/pnas.1302880110 PMC3696826 23759743

[b22] Larger L., Penkovsky B. & Maistrenko Y. L. Virtual chimera states for delayed-feedback systems Phys. Rev. Lett. 111, 054103 (2013). 23952404 10.1103/PhysRevLett.111.054103

[b23] Larger L., Penkovsky B. & Maistrenko Yu. Laser chimeras as a paradigm for multistable patterns in complex systems. Nat. Commun. 6, 7752 (2015). 26169585 10.1038/ncomms8752 PMC4510973

[b24] Kapitaniak T., Kuzma. P., Wojewoda J., Czolczynski K. & Maistrenko Yu. Imperfect chimera states for coupled pendula. Sci. Rep. 4, 6379 (2014). 25223296 10.1038/srep06379 PMC5376200

[b25] Olmi S., Martens E. M., Thutupalli S. & Torcini A. Intermittent chaotic chimeras for coupled rotators. Phys. Rev. E 92, 03090 (R) (2015). 10.1103/PhysRevE.92.030901 26465413

[b26] Panaggio M. & Abrams D. 2015 Chimera states: coexistence of coherence and incoherence in networks of coupled oscillators. Nonlinerity. 28, 67–87 (2015).

[b27] Ashwin P. & Burylko O. Weak chimeras in minimal networks of coupled phase oscillators. Chaos 25, 013106 (2015). 25637917 10.1063/1.4905197

[b28] Panaggio M. J., Abrams D. M., Ashwin P. & Laing C. Chimera states in networks of phase oscillators: the case of two small populations. Phys. Rev. E 93, 012218 (2016). 26871084 10.1103/PhysRevE.93.012218

[b29] Bick Ch. & Aswin P. Chaotic weak chimeras and their persistence in coupled populations of phase oscillators. Nonlinearity 29, 1468 (2016).

[b30] Strzalko J., Grabski J., Wojewoda J., Wiercigroch M. & Kapitaniak T. Synchronous rotation of the set of double pendula: Experimental observations. Chaos 22, 047503 (2012). 23278089 10.1063/1.4740460

[b31] Nizhnik L. P., Nizhnik I. L. & Hasler M. Stable stationary solutions in reaction-diffusion systems consisting of a 1-D array of bistable cells. Int. J. Bifurcation Chaos 12, 261 (2002).

[b32] Doedel E. J. & Oldeman B. E. Auto-07P: Continuation and Bifurcation Software for Ordinary Differential Equations, Concordia University, Montreal, Canada, 2009.

