# Supplementary material for: Experimental multistable states for small network of coupled pendula
Source: Sci Rep. 2016 Jul 21;6:29833. doi: 10.1038/srep29833 (PMC4956754; doi:10.1038/srep29833)
Supplement: Supplementary Information [file srep29833-s10.pdf]

Experimental multistable states for small network of coupled pendula

Dawid Dudkowski[1], Juliusz Grabski[1], Jerzy Wojewoda[1], Przemyslaw Perlikowski[1],

Yuri Maistrenko[1,2,3], & Tomasz Kapitaniak[1*]

[1]Division of Dynamics, Technical University of Lodz, Stefanowskiego 1/15, 90-924 Lodz, Poland,
[2]Institute of Mathematics and Centre for Medical and Biotechnical Research, National Academy of Sciences of Ukraine, Tereshchenkivska st. 3, 01030 Kyiv, Ukraine
[3]Institut fur Theoretische Physik, Technische Universitat Berlin, Hardenbergstrasse 36, 10623 Berlin, Germany

Supplementary information

Movie W1. Multistable chimera-like state: pendula 1 and 2 rotate with a frequency $\frac{1}{3}\omega$, pendula 3 and 4 with frequency $\frac{1}{2}\omega$.

Movie W2. Multistable chimera-like state: pendula 1,3 and 4 rotate with frequency $\frac{1}{2}\omega$ and pendulum 2 with frequency $\frac{1}{3}\omega$.

Movie W3. Multistable chimera-like state: pendulum 1 rotates with a frequency $\frac{1}{3}\omega$, pendula 2 and 3 with frequency ω and pendulum 4 with frequency $\frac{1}{2}\omega$.

Movie W4. Multistable chimera-like state: pendula 1, 2 and 4 rotate with frequency $\frac{1}{3}\omega$ and pendulum 3 with frequency ω.

Movie W5. Multistable chimera-like state: pendula 1,3 and 4 rotate with the frequency $\frac{1}{2}\omega$, pendulum 2 oscillates with the frequency $\frac{1}{2}\omega$.

Movie W6. Multistable chimera-like state: pendula 1 and 4 rotate with the frequency ω, pendulum 2 rotates with the frequency $\frac{1}{2}\omega$ and pendulum 3 oscillates with the frequency $\frac{1}{2}\omega$
.

Movie W7. Synchronous state: all pendula rotate with the frequency ω and are synchronized.

Movie W8. Synchronous state: all pendula oscillate with the frequency ω, pendula 2,3 and 4 are synchronized in phase and pendulum 1 is in antiphase to them.

Movie W9. Synchronous state: all pendula oscillate with the frequency ω, two clusters synchronized with antiphase.

Movies W1-4 and W7: A=0.01[m], ω=18π [rad/s] (region 5 of Figure 2),

Movies W5-6 and W8-9: A=0.005[m], ω=10π [rad/s] (region 1 of Figure 2).
